# Women's Rugby League: Positional Groups and Peak Locomotor Demands

**DOI:** 10.3389/fspor.2021.648126

**Published:** 2021-06-29

**Authors:** Cloe Cummins, Glen Charlton, David Paul, Kath Shorter, Simon Buxton, Johnpaul Caia, Aron Murphy

**Affiliations:** ^1^School of Science and Technology, University of New England, Armidale, NSW, Australia; ^2^National Rugby League, Sydney, NSW, Australia; ^3^Carnegie Applied Rugby Research Centre, Carnegie School of Sport, Leeds Beckett University, Leeds, United Kingdom; ^4^Faculty of Medicine, Nursing and Midwifery and Health Sciences, University of Notre Dame, Fremantle, WA, Australia

**Keywords:** female athlete, GPS, match demands, microtechnology, team sport

## Abstract

The aims of this study were to (a) use a data-based approach to identify positional groups within National Rugby League Women's (NRLW) match-play and (b) quantify the peak locomotor demands of NRLW match-play by positional groups. Microtechnology (Global Navigational Satellite System [GNSS] and integrated inertial sensors; *n* = 142 files; *n* = 76 players) and match statistics (*n* = 238 files; *n* = 80 players) were collected from all NRLW teams across the 2019 season. Data-based clustering of match statistics was utilized to identify positional clusters through classifying individual playing positions into distinct positional groups. Moving averages (0.5, 1, 2, 3, 5, and 10 min) of peak running and average acceleration/deceleration demands were calculated via microtechnology data for each player per match. All analysis was undertaken in R (R Foundation for Statistical Computing) with positional differences determined via a linear mixed model and effect sizes (ES). Data-based clustering suggested that, when informed by match statistics, individual playing positions can be clustered into one of three positional groups. Based on the clustering of the individual positions, these groups could be broadly defined as backs (fullback, wing, and center), adjustables (halfback, five-eighth, and hooker), and forwards (prop, second-row, and lock). Backs and adjustables demonstrated greater running (backs: ES 0.51–1.00; *p* < 0.05; adjustables: ES 0.51–0.74, *p* < 0.05) and average acceleration/deceleration (backs: ES 0.48–0.87; *p* < 0.05; adjustables: ES 0.60–0.85, *p* < 0.05) demands than forwards across all durations. Smaller differences (small to trivial) were noted between backs and adjustables across peak running and average acceleration/deceleration demands. Such findings suggest an emerging need to delineate training programs in situations in which individual playing positions train in positional group based settings. Collectively, this work informs the positional groupings that could be applied when examining NRLW data and supports the development of a framework for specifically training female rugby league players for the demands of the NRLW competition.

## Introduction

Recently, there has been an increase in the participation rates, playing opportunities, and professionalization of female rugby league (Cummins et al., 2020b). For example, at the elite level, a women's premiership competition (National Rugby League Women's [NRLW]; inaugural year: 2018) was recently established by the National Rugby league (NRL). The NRLW is structured via a round robin format, whereby each of the teams (*n* = 4) play each other once. At the completion of the regular season (round robin), the two highest ranking teams contest the grand final in order to determine the overall premiership winner. The NRLW is governed by similar rules as the men's NRL competition with matches being played on the standard full-size field (~68 m wide x 120 m long [including the in-goal area]). There are, however, several differences between NRLW and NRL matches. Specifically, NRLW matches are shorter in duration than that of the NRL competition with matches being played over halves of 30 min (plus additional time allocated for stoppages). Furthermore, the NRLW competition includes the 40/30 kick advantage and permits more interchanges than the men's NRL competition (10 vs. 8 interchanges per match, respectively).

The use of microtechnology (Global Navigational Satellite System [GNSS] and integrated inertial sensors) has provided an enhanced understanding of the demands of team sports (Cummins et al., 2013). Although such devices have been used extensively to understand the locomotor demands (i.e., the physical demands associated with motion, for instance, this may include walking, running, or sprinting) of men's rugby league (Johnston et al., 2014; Hausler et al., 2016; Glassbrook et al., 2019), comparatively less is understood about women's rugby league (Cummins et al., 2020a; Emmonds et al., 2020; Quinn et al., 2020; Newans et al., 2021). Although the aforementioned research reports on automated tackle detection (Cummins et al., 2020a), the peak (Emmonds et al., 2020) and whole match (Emmonds et al., 2020; Quinn et al., 2020) locomotor demands of international teams, the whole and peak locomotor demands of the Women's Super League (WSL) competition (Emmonds et al., 2020), and the whole match locomotor demands of the NRLW competition (Newans et al., 2021), to the authors' knowledge, no research exists on the peak locomotor demands of the NRLW competition.

Examination of match characteristics provides an understanding of the locomotor demands of rugby league players. The intermittent nature of the game, however, means that such analysis may not truly represent the highly variable intensity of match-play (Whitehead et al., 2018). For example, English international female backs cover an average of 75.2 m.min^−1^ throughout a match, and the same players cover an average of 144, 93, and 81 m.min^−1^ across 1-, 5-, and 10-min durations, respectively (Emmonds et al., 2020). This equates to 68.8, 17.8, and 5.8 m.min^−1^ more than the overall match intensity, respectively (Emmonds et al., 2020). Due to such discrepancies, examining locomotor demands across duration-specific time periods is important in enabling practitioners to develop training programs that specifically prepare players for the maximum demands and intensities of match-play.

Additionally, there is an inconsistency in the classification of positional groups throughout rugby league research (Cummins et al., 2013). Research within the women's game specifically has reported on the locomotor demands of match-play by each position (Newans et al., 2021) as well as by two (i.e., forwards and backs) (Emmonds et al., 2020) and three (i.e., backs, halves, and forwards) (Quinn et al., 2020) positional groups. Due to the disparities in the physical qualities of male (Johnston et al., 2014) and female players (Jones et al., 2016) and the evolving nature of women's rugby league, it is possible that such positional groups may not represent the true positional groupings and, therefore, not reflect the actual demands of female rugby league players.

Collectively, the paucity of research on the demands of women's rugby league hinders the development of a framework to inform female-specific development, training, and management practices (Cummins et al., 2020b). Therefore, the aims of this study were to (a) use a data-based approach to identify positional groups within NRLW match-play and (b) quantify the peak locomotor demands of NRLW match-play by positional groups.

## Materials and Methods

### Participants

Match-play data were collected from professional female rugby league players representing all NRLW teams (*n* = 4; *n* = 80 players) over one competitive season (2019). Institutional ethics approval was granted by the University of New England Human Research Ethics Committee.

### Microtechnology Data Collection

Microtechnology data (*n* = 142 files; *n* = 76 players) were captured via microtechnology devices (OptimEye S5; Catapult Sports Melbourne, Australia), which record a 10 Hz GNSS sampling rate through the inbuilt GNSS-chip. Each NRLW team was responsible for collecting and downloading microtechnology data from each match with the corresponding raw data being utilized in the calculation of peak locomotor demands.

### Data Manipulation

A database was created that contained the 10 Hz GNSS data (Catapult Sports Melbourne, Australia) and match-play statistics (Stats Perform, Chicago, Illinois, United States) as retrieved from the RLeague Analyser (Fair Play, Jindalee, Queensland, Australia).

The microtechnology and match-play statistics data sets were synchronized in order to apply the start and end times of each half per match (and extra time) as well as the interchange times for each player. Erroneous data within a file was flagged if any of the following criteria was met: (a) acceleration >6 m.s^−2^ (Weston et al., 2015), (b) velocity >10 m.s^−1^ (Weston et al., 2015), or (c) a traveled distance of >10 m in a 1 s time period. This combination of criteria ensured that erroneous velocity and distance data calculated via either the doppler-shift or positional differentiation methods were identified (Malone et al., 2017). Similar to common practice within high-performance settings, any period of erroneous data (including 1 s of data on either side) was removed and, therefore, excluded from analysis.

From the initial 142 microtechnology files, files were removed due to poor signal quality, whereby more than 5% of the raw data was flagged as erroneous and removed, or a total match duration of less than a quarter (i.e., 15 min, which is equivalent to 20 min, within men's rugby league; Dalton-Barron et al., 2020). A total of 131 files were included in the analysis.

### Locomotor Variables

To calculate duration-specific peak average running demands (m.min^−1^), each players' instantaneous velocity was used in a custom-built algorithm (R Foundation for Statistical Computing, Vienna, Austria; version 4.02) to calculate a moving average of instantaneous running speed (m.min^−1^) (Delaney et al., 2015; Weaving et al., 2018) across six different durations (0.5, 1, 2, 3, 5, and 10 min) for each match. The peak average acceleration/deceleration (m.s^−2^) was calculated as the rate of change in velocity regardless of direction (Delaney et al., 2016). This was achieved through averaging the absolute value of all acceleration and deceleration data across defined periods (Delaney et al., 2016) of six different durations (0.5, 1, 2, 3, 5, and 10 min) for each match. Within the context of load monitoring, this measure provides insight into the overall acceleration/deceleration load experienced by an athlete and can be utilized to inform training prescription (Delaney et al., 2016). This measure is shown to demonstrate increased interunit reliability when compared with the use of threshold-based acceleration measures (Delaney et al., 2018). Specifically, when multiple 10 Hz devices were attached to a sprint sled throughout a team sport simulation protocol, the interunit reliability of the average acceleration/deceleration measure was 1.2% (coefficient of variation; CV), and the interunit reliability ranged from 3.3 to 5.9% (CV) across intensity-based thresholds (Delaney et al., 2018). It is also suggested that, although a 10 Hz device can determine whether an acceleration or deceleration has occurred, there is a degree of error in the measurement of instantaneous velocity (Varley et al., 2012). This suggests that the average acceleration/deceleration measure may be more appropriate than threshold-based measures in monitoring the acceleration demands of team sport athletes (Delaney et al., 2018).

### Positional Groups

The parameterized finite mixture model algorithm from the “mclust package” (Fraley and Raftery, 1999) was used on match statistics data (*n* = 238 files; *n* = 80 players) to classify individuals into distinct positional groups. This algorithm is designed to take independent identically distributed observations and provide model-based hierarchical clustering, heuristically calculating probabilities (percentage) that each independent player's observations belong to a particular classification, attempting to realize wider population patterns based on the limited sample data. The variables of minutes played, hit-ups, run meters (i.e., meters run in possession of the ball), line breaks, kicks, tackles, and passes were utilized based on their relationship to playing style and the authors' expertise in women's rugby league. Although these variables are not completely independent (e.g., a player on the field for fewer minutes is likely to have reduced match statistics), the algorithm still has potential to offer insight into positional groups even if results cannot be conclusive. The individual positions were partitioned until the algorithm could make no further grouping with each position clustered into the grouping containing the majority of data points.

### Statistical Analysis

All statistical analyses were undertaken in R (R Foundation for Statistical Computing, Vienna, Austria; version 4.0.2). Data are presented as mean ± 90% confidence intervals (CI) unless otherwise stated. Positional group differences were compared using a linear mixed model with Bonferroni *post-hoc* testing. The level of significance was accepted at *p* ≤ 0.05. Cohen effect size (ES) with 90% CI were also used to examine differences between playing positions. Effect sizes were categorized as trivial (<0.2), small (0.2–0.6), moderate (>0.6–1.2), large (>1.2–2.0), or very large (>2.0) (Hopkins et al., 2009).

## Results

**Figure 1:**
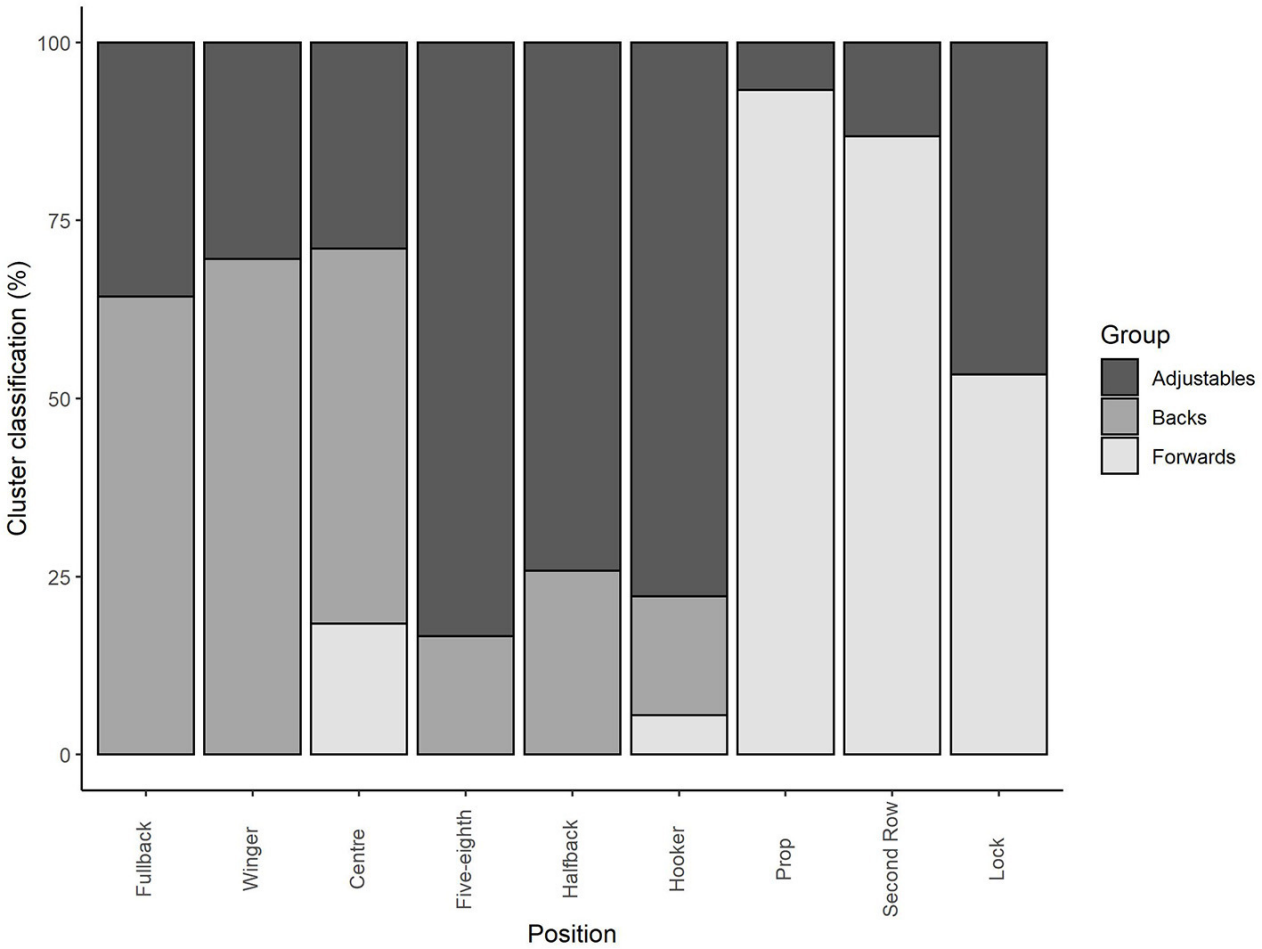
Cluster classification of positional groups.

As seen in Figure 1, data-based clustering classified players into one of three groups: 1 (fullback, wing, and center), 2 (halfback, five-eighth, and hooker), and 3 (prop, second-row, and lock). The figure shows, for example, that 52.6% of centers are clustered in group 1, 28.9% are clustered into group 2, and 18.4% are clustered into group 3. Based on the clustering of the individual positions, these groupings could be broadly defined as backs (group 1), adjustables (group 2), and forwards (group 3). The probability (represented as a percentage) of each position belonging to the respective group was 52.6–69.6% for backs, 74.2–83.3% for adjustables, and 53.3–93.3% for forwards.

Backs and adjustables demonstrated greater peak running (backs: ES 0.51–1.00; *p* ≤ 0.05; adjustables: ES 0.51–0.74, *p* ≤ 0.05) and average acceleration/deceleration (backs: ES 0.48–0.87; *p* ≤ 0.05; adjustables: ES 0.60–0.85, *p* ≤ 0.05) demands than forwards across all durations (Tables 1, 2). Smaller differences (trivial to small) were noted in the peak running and acceleration demands between backs and adjustables (Tables 1, 2).

**Table 1:** Peak running demands.

	**Adjustables (m.min^**−1**^)**	**Backs (m.min^**−1**^)**	**Forwards (m.min^**−1**^)**	**Adjustables vs. Backs (ES ± 90% CI)**	**Adjustables vs. Forwards (ES ± 90% CI)**	**Backs vs. Forwards (ES ± 90% CI)**
0.5 min	179.6 ± 7.5	188.8 ± 8.2	161.1 ± 5.3	−0.31 ± 0.38	0.74 ± 0.37^*^	1.00 ± 0.36^*^
1 min	149.1 ± 6.0	147.6 ± 5.4	136.9 ± 4.4	0.07 ± 0.38	0.60 ± 0.37^*^	0.53 ± 0.34^*^
2 min	121.7 ± 5.0	121.1 ± 4.3	112.0 ± 3.6	0.03 ± 0.38	0.58 ± 0.37^*^	0.56 ± 0.35^*^
3 min	111.6 ± 4.2	110.7 ± 3.7	102.3 ± 3.4	0.06 ± 0.38	0.62 ± 0.37^*^	0.56 ± 0.35^*^
5 min	99.9 ± 4.3	99.1 ± 3.0	92.5 ± 3.2	0.06 ± 0.37	0.51 ± 0.36^*^	0.51 ± 0.35^*^
10 min	88.6 ± 4.3	89.1 ± 2.6	81.0 ± 3.1	−0.04 ± 0.38	0.53 ± 0.36^*^	0.66 ± 0.35^*^

*Data presented as mean ± 90% confidence interval (unless otherwise stated); CI, confidence interval; ES, effect size;*

* *, significant difference (P ≤ 0.05)*.

**Table 2:** Average acceleration/deceleration demands.

	**Adjustables (m.s^**−2**^)**	**Backs (m.s^**−2**^)**	**Forwards (m.s^**−2**^)**	**Adjustables vs. Backs (ES ± 90% CI)**	**Adjustables vs. Forwards (ES ± 90% CI)**	**Backs vs. Forwards (ES ± 90% CI)**
0.5 min	0.92 ± 0.03	0.95 ± 0.02	0.87 ± 0.02	−0.26 ± 0.38	0.60 ± 0.37^*^	0.87 ± 0.36^*^
1 min	0.77 ± 0.02	0.77 ± 0.02	0.72 ± 0.02	0.04 ± 0.38	0.62 ± 0.37^*^	0.60 ± 0.35^*^
2 min	0.64 ± 0.02	0.63 ± 0.01	0.60 ± 0.01	0.27 ± 0.37	0.67 ± 0.37^*^	0.48 ± 0.35^*^
3 min	0.60 ± 0.02	0.58 ± 0.01	0.55 ± 0.01	0.41 ± 0.38	0.84 ± 0.37^*^	0.57 ± 0.35^*^
5 min	0.54 ± 0.02	0.52 ± 0.01	0.49 ± 0.01	0.36 ± 0.38	0.85 ± 0.38^*^	0.66 ± 0.35^*^
10 min	0.49 ± 0.02	0.47 ± 0.01	0.43 ± 0.01	0.26 ± 0.37	0.83 ± 0.37^*^	0.76 ± 0.35^*^

*Data presented as mean ± 90% confidence interval (unless otherwise stated); CI, confidence interval; ES, effect size;*

* *, significant difference (P ≤ 0.05)*.

## Discussion

To the authors' knowledge, this is the first study to examine the positional groups and peak locomotor demands of NRLW match-play. The findings identify that, within this data set, individual playing positions can be clustered into three positional groups (i.e., backs, adjustables, and forwards) and that, when compared with forwards, both backs and adjustables demonstrate increased peak running and average acceleration/deceleration demands. Together, this work informs the positional groupings that could be applied when examining NRLW data and supports the development of a framework for specifically training female rugby league players for the demands of the NRLW competition.

Data-based clustering suggests that, when informed by match statistics, individual playing positions can be clustered into one of three positional groups (Figure 1). Based on the clustering of the individual positions, these groupings could be defined as backs (fullback, wing, and center), adjustables (halfback, five-eighth, and hooker), and forwards (prop, second-row, and lock). The differences in classifications indicate that, irrespective of their named position (e.g., center), individual players undertake specific roles on the field. For example, although the majority of centers were classified as a back, 28.9% and 18.4% were classified as adjustables or forwards, respectively. Such findings suggest that some centers within women's rugby league undertake a more ball playing (e.g., adjustable) or defensive (e.g., forward) role on the field. For the forward position, although the positions of prop (93.3%) and second row (86.8%) distinctly fell into the category of a forward, the lock was categorized as a forward (53.3%) or adjustable (46.7%), suggesting that, across the NRLW, some teams may utilize a lock player in a more ball-playing role. The exhibited variances in positional classifications across the three groups could be attributable to a myriad of factors, including team tactics/game plays, whereby the coaching or playing style across the four teams competing within the NRLW may influence the role undertaken by individual players. Additionally, these variances may reflect the evolving nature of women's rugby league, whereby as the data was gleaned from the second NRLW season, it is plausible that female rugby league players are yet to differentiate into distinct positions or that players may rotate between positions (Clarke et al., 2018). Similarly, within the inaugural year of the Australian Football League Women's competition, it was reported that there may not have been sufficient time for the development of distinct technical and tactical demands across playing positions (Clarke et al., 2018). Conversely, it may be that female rugby league players demonstrate a more homogenous style of play across playing positions. It should also be noted that microtechnology data was unable to cluster individual positions into clear groups. Despite the aforementioned variances within the positional classifications, the findings are supportive of an emerging need to delineate training programs in situations in which individual playing positions train in positional group based settings. Further, this informs the positional groupings that could be applied when examining or conducting research on NRLW data that is gleaned from one team where the influence of individual players (i.e., one fullback) reduces the translation of position-specific findings across teams more broadly. Further work is warranted to elucidate whether these groupings change with additional NRLW seasons as well as the positional groupings that could be utilized across other female rugby league competitions, such as the WSL.

Positional group differences were apparent with backs and adjustables demonstrating increased peak running and average acceleration/deceleration demands than forwards (Tables 1, 2). Conversely, backs and adjustables demonstrated close similarity in peak running and average acceleration/deceleration demands. Previous work suggests that practical differences (i.e., the smallest threshold that can be translated into both prescription and monitoring) can be observed via a threshold of 10 m.min^−1^ (Delaney et al., 2015). Based upon this, there is no practical difference between the peak running demands of backs and adjustables across the reported duration-specific time points, thereby indicating that, although position-specific training intensities may be required between backs/adjustables and forwards, it may not be necessary to train adjustables and backs separately in regards to this metric. The increased average acceleration/deceleration profile of adjustables and backs when compared with forwards suggests that, alongside maintaining an increased running intensity, they are engaged in more start/stop actions. This suggests that backs and adjustables should undertake training and conditioning drills that replicate these demands through a focus on sustained intensity as well as accelerations/decelerations and changes of direction. The ability to compare such findings to previous work is hindered through differences in playing durations, whereby NRLW matches are 60 min in duration and WSL matches are 80 min in duration (Emmonds et al., 2020), the different positional groupings across studies (Emmonds et al., 2020; Quinn et al., 2020; Newans et al., 2021) as well as the reporting of different locomotor metrics (Quinn et al., 2020; Newans et al., 2021).

It should be acknowledged that the relatively small number of teams (*n* = 4) competing within the NRLW means that the team tactics/game plays and preferences of the coaching staff could have an influence upon the positional groupings and locomotor demands that were elucidated within this work. Despite this potential and unavoidable limitation, the findings of this work inform the positional groupings that could be applied to the analysis of NRLW data and the development of a framework to support female-specific training programs that prepare players for the peak demands of match-play. Future work should look to review the positional groups and peak demands of NRLW match-play as the game continues to develop.

## Conclusion

This study suggests that three positional groups (i.e., backs, adjustables, and forwards) exist and that, when compared with forwards, both backs and adjustables demonstrate increased peak running and average acceleration/deceleration demands. Such findings suggest that backs and adjustables should undertake training and conditioning drills that replicate increased peak running and average acceleration/deceleration demands through a focus on sustained intensity as well as accelerations/decelerations and changes of direction. Collectively, this work informs the positional groupings that could be applied when examining NRLW data and supports the development of a framework for specifically training female rugby league players for the demands of the NRLW competition.

## Data Availability Statement

The data sets presented in this article are not readily available because the authors do not have permission to share the respective datasets. Requests to access the data set should be directed to cloe.cummins@une.edu.au.

## Ethics Statement

The studies involving human participants were reviewed and approved by the University of New England human research ethics committee. Written informed consent for participation was not required for this study in accordance with the national legislation and the institutional requirements.

## Author Contributions

CC: conceptualization, design, and original drafting of the manuscript. JC: data collection. CC, GC, and DP: data interpretation and analysis. CC, GC, DP, KS, SB, JC, and AM: critical revision of the manuscript. All authors contributed to the article and approved the submitted version.

## Conflict of Interest

At the time of submission JC and SB held employment with the National Rugby League. The National Rugby League provided support in the form of research funding for CC. The remaining authors declare that the research was conducted in the absence of any commercial or financial relationships that could be construed as a potential conflict of interest.
